# Supplementary material for: Fine Mapping and Functional Analysis of Major Regulatory Genes of Soluble Solids Content in Wax Gourd (Benincasa hispida)
Source: Int J Mol Sci. 2022 Jun 23;23(13):6999. doi: 10.3390/ijms23136999 (PMC9266771; doi:10.3390/ijms23136999)
Supplement: Supplementary file 1 [file ijms-23-06999-s001.zip › ijms-1782727-supplementary.pdf]

# *Supplementary Material*

**Supplementary Table S1.** Primers used in study

| Primer ID | Forward sequence(5′-3′) | Reverse sequence(5′-3′) | Application |
|---|---|---|---|
| qPCR-NC | TACCCCATGCTATCCTCCGT | TTCCTGCTCATAGTCGAGCG | RT-qPCR for Bch10G018990 |
| qPCR-85 | CAATTTGATTCTCAATCTGACCTCC | TGGGTCTTCAAAAGATGATAAGAGG | RT-qPCR for Bch02G016850 |
| qPCR-86 | AGTACATTTACTGCCTATGAAGCTG | CGTCAAAAGAGTCCCGACA | RT-qPCR for Bch02G016860 |
| qPCR-87 | CTACATTTACTGCCTATGAAGC | GAATCTGATTGTTACCGTCG | RT-qPCR for Bch02G016870 |
| qPCR-88 | GCATCGAATGATGGATTTGA | AAGTCCGTCCTTTGGCTCT | RT-qPCR for Bch02G016880 |
| qPCR-89 | TTTTGCTCCGATTGAACCA | AATGAAAGGGGGGAAGGTT | RT-qPCR for Bch02G016890 |
| qPCR-90 | TCTCAAGCCGTCCGAATGA | TAAGCCGCCAGTAGCAGCA | RT-qPCR for Bch02G016900 |
| qPCR-91 | TAAATACGGGGACACGTCC | TCTGGAAAACAATGGCGA | RT-qPCR for Bch02G016910 |
| qPCR-92 | TAAATACGGGGACACGTCC | TCTGGAAAACAATGGCGA | RT-qPCR for Bch02G016920 |
| qPCR-93 | CTTCTCTCTTAGCAATGCCT | CTATTACCAGCTCGAACTCTC | RT-qPCR for Bch02G016930 |
| qPCR-94 | AGACGACGTTTCCCTTGCT | CTGCTGCTGTTTTGCTCCA | RT-qPCR for Bch02G016940 |
| qPCR-95 | AAAGGAGTCTGTGGTAGACAAGGAT | TCCTTTTCTTTCGCTCGCC | RT-qPCR for Bch02G016950 |
| qPCR-96 | CTCCGACAACGACTTCACA | TATAGCAGCCTCCGCTACA | RT-qPCR for Bch02G016960 |
| qPCR-97 | TGTTGGACTTGGACCCGAA | AGCCCCCGCAAATAATGAG | RT-qPCR for Bch02G016970 |
| KL-96 | ATGGAGAGTACTTTGAAGGAGATCG | TGTACATGCAGCTCTCAGCATACTT | CDS of Bch02G016960 |
| InDel-96 | TCATAGATTTGATTTCAGGGCA | CAGTATCATTGAATTCGCACG | marker for molecular marker-assisted breeding |

```
GX-19.txt     ATGGAGAGTACTTTGAAGGAGATCGGCGATGGCGGCTCAGTGTTGGACTTGGACCCGAAAGCCACCGTTGGCGGTGGTGTTGAGGATATCTACGGAGAAGACTGTGCTAC   110
SL-7.txt      ATGGAGAGTACTTTGAAGGAGATCGGCGATGGCGGCTCAGTGTTGGACTTGGACCCGAAAGCCACCGTTGGCGGTGGTGTTGAGGATATCTACGGAGAAGACTGTGCTAC   110
XDJQ-1.txt    ATGGAGAGTACTTTGAAGGAGATCGGCGATGGCGGCTCAGTGTTGGACTTGGACCCGAAAGCCACCGTTGGCGGTGGTGTTGAGGATATCTACGGAGAAGACTGTGCTAC   110
Consensus     atggagagtactttgaaggagatcggcgatggcggctcagtgttggacttggacccgaaagccaccgttggcggtggtgttgaggatatctacggagaagactgtgctac

GX-19.txt     AGAGGAGCAGCTTGTTACTCCATGGACTTTCTCAGTTGCGAGTGGCTATTCGTTGTTGAGGGATCCTCACCATAACAAGGGGTTGGCATTTACAGAGAAGGAAAGAGATG   220
SL-7.txt      AGAGGAGCAGCTTGTTACTCCATGGACTTTCTCAGTTGCGAGTGGCTATTCGTTGTTGAGGGATCCTCACCATAACAAGGGGTTGGCATTTACAGAGAAGGAAAGAGATG   220
XDJQ-1.txt    AGAGGAGCAGCTTGTTACTCCATGGACTTTCTCAGTTGCGAGTGGCTATTCGTTGTTGAGGGATCCTCACCATAACAAGGGGTTGGCATTTACAGAGAAGGAAAGAGATG   220
Consensus     agaggagcagcttgttactccatggactttctcagttgcgagtggctattcgttgttgagggatcctcaccataacaaggggttggcatttacagagaaggaaagagatg

GX-19.txt     CTCATTATTTGCGGGGGCTTCTGCCACCTGCCATCGTTACTCAACAACTTCAGGAGAAGAAGTTGATGCAGAACATCAGGCAGTATCAACTTCCACTACAAAAATTTATT   330
SL-7.txt      CTCATTATTTGCGGGGGCTTCTGCCACCTGCCATCGTTACTCAACAACTTCAGGAGAAGAAGTTGATGCAGAACATCAGGCAGTATCAACTTCCACTACAAAAATTTATT   330
XDJQ-1.txt    CTCATTATTTGCGGGGGCTTCTACCACCTGCCATCGTTACTCAACAACTTCAGGAGAAGAAGTTGATGCAGAACATCAGGCAGTATCAACTTCCACTACAAAAATTTATT   330
Consensus     ctcattatttgcgggggcttct ccacctgccatcgttactcaacaacttcaggagaagaagttgatgcagaacatcaggcagtatcaacttccactacaaaaatttatt

GX-19.txt     GCCATGATGGAACTTCAGGAAAGAAATGAAAGGCTTTTTTACAAACTTCTTATTGACAACGTCGAAGAACTGCTTCCTGTCGTCTACACTCCCACAGTTGGTGAAGCTTG   440
SL-7.txt      GCCATGATGGAACTTCAGGAAAGAAATGAAAGGCTTTTTTACAAACTTCTTATTGACAACGTCGAAGAACTGCTTCCTGTCGTCTACACTCCCACAGTTGGTGAAGCTTG   440
XDJQ-1.txt    GCCATGATGGAACTTCAGGAAAGAAATGAAAGGCTTTTTTACAAACTTCTTATTGACAACGTCGAAGAACTGCTTCCTGTCGTCTACACTCCCACAGTTGGTGAAGCTTG   440
Consensus     gccatgatggaacttcaggaaagaaatgaaaggcttttttacaaacttcttattgacaacgtcgaagaactgcttcctgtcgtctacactcccacagttggtgaagcttg

GX-19.txt     TCAGAAGTATGGAAGCATCTTTAGGCGTCCTCAGGGTCTTTACATCAGTTTGAAAGAGAAGGGTAAGATTCTTGAAGTATTGAAGAACTGGCCCCAAAGGAGCATCCAAG   550
SL-7.txt      TCAGAAGTATGGAAGCATCTTTAGGCGTCCTCAGGGTCTTTACATCAGTTTGAAAGAGAAGGGTAAGATTCTTGAAGTATTGAAGAACTGGCCCCAAAGGAGCATCCAAG   550
XDJQ-1.txt    TCAGAAGTATGGAAGCATCTTTAGGCGTCCTCAGGGTCTTTACATCAGTTTGAAAGAGAAGGGTAAGATTCTTGAAGTATTGAAGAACTGGCCCCAAAGGAGCATCCAAG   550
Consensus     tcagaagtatggaagcatctttaggcgtcctcagggtctttacatcagtttgaaagagaagggtaagattcttgaagtattgaagaactggccccaaaggagcatccaag

GX-19.txt     TAATTGTGGTGACTGATGGTGAGCGTATTTTGGGTCTTGGTGATCTTGGTTGTCAGGGAATGGGAATTCCAGTTGGGAAACTTTCTTTATACACCGCACTTGGAGGAGTT   660
SL-7.txt      TAATTGTGGTGACTGATGGTGAGCGTATTTTGGGTCTTGGTGATCTTGGTTGTCAGGGAATGGGAATTCCAGTTGGGAAACTTTCTTTATACACCGCACTTGGAGGAGTT   660
XDJQ-1.txt    TAATTGTGGTGACTGATGGTGAGCGTATTTTGGGTCTTGGTGATCTTGGTTGTCAGGGAATGGGAATTCCAGTTGGGAAACTTTCTTTATACACCGCACTTGGAGGAGTT   660
Consensus     taattgtggtgactgatggtgagcgtattttgggtcttggtgatcttggttgtcagggaatgggaattccagttgggaaactttctttatacaccgcacttggaggagtt

GX-19.txt     CGTCCTTCTGCATGTTTGCCTATTACAATTGATGTTGGGACAAACAACGAGAAATTGTTGAATGATGAATTCTACATTGGGCTTAAACAGAGAAGAGCGACTGGAGAGGA   770
SL-7.txt      CGTCCTTCTGCATGTTTGCCTATTACAATTGATGTTGGGACAAACAACGAGAAATTGTTGAATGATGAATTCTACATTGGGCTTAAACAGAGAAGAGCGACTGGAGAGGA   770
XDJQ-1.txt    CGTCCTTCTGCATGTTTGCCTATTACAATTGATGTTGGGACAAACAACGAGAAATTGTTGAATGATGAATTCTACATTGGGCTTAAACAGAGAAGAGCGACTGGAGAGGA   770
Consensus     cgtccttctgcatgtttgcctattacaattgatgttgggacaaacaacgagaaattgttgaatgatgaattctacattgggcttaaacagagaagagcgactggagagga

GX-19.txt     GTACTATGAACTTCTAGATGAGTTTATGACTGCTGTTAAGCAGAATTATGGGGAAAAGGTTCTGGTTCAGTTCGAAGATTTTGCAAACCACAATGCTTTTGAGCTGCTCG   880
SL-7.txt      GTACTATGAACTTCTAGATGAGTTTATGACTGCTGTTAAGCAGAATTATGGGGAAAAGGTTCTGGTTCAGTTCGAAGATTTTGCAAACCACAATGCTTTTGAGCTGCTCG   880
XDJQ-1.txt    GTACTATGAACTTCTAGATGAGTTTATGACTGCTGTTAAGCAGAATTATGGGGAAAAGGTTCTGGTTCAGTTCGAAGATTTTGCAAACCACAATGCTTTTGAGCTGCTCG   880
Consensus     gtactatgaacttctagatgagtttatgactgctgttaagcagaattatggggaaaaggttctggttcagttcgaagattttgcaaaccacaatgcttttgagctgctcg

GX-19.txt     CCAAGTATCGCACAACTCATCTAGTCTTCAACGATGATATCCAGGGGACAGCAGCTGTCGTACTTGCTGGAGCTGTTTCTGCCCTCAAACTTATTGGTGGTACCTTGGCC   990
SL-7.txt      CCAAGTATCGCACAACTCATCTAGTCTTCAACGATGATATCCAGGGGACAGCAGCTGTCGTACTTGCTGGAGCTGTTTCTGCCCTCAAACTTATTGGTGGTACCTTGGCC   990
XDJQ-1.txt    CCAAGTATCGCACAACTCATCTAGTCTTCAACGATGATATTCAGGGGACAGCAGCTGTCGTACTTGCTGGAGCTGTTTCTGCCCTCAAACTTATTGGTGGTACCTTGGCC   990
Consensus     ccaagtatcgcacaactcatctagtcttcaacgatgatat caggggacagcagctgtcgtacttgctggagctgtttctgccctcaaacttattggtggtaccttggcc

GX-19.txt     GATCACACGTTCTTGTTCCTTGGTGCTGGGGAAGCTGGAACTGGTATTGCAGAGCTTATAGCCCTTGAAGTATCAAAACAGACAAATGCTCCAGTTGAGGAGACGCGCAA   1100
SL-7.txt      GATCACACGTTCTTGTTCCTTGGTGCTGGGGAAGCTGGAACTGGTATTGCAGAGCTTATAGCCCTTGAAGTATCAAAACAGACAAATGCTCCAGTTGAGGAGACGCGCAA   1100
XDJQ-1.txt    GATCACACATTCTTGTTCCTTGGTGCTGGGGAAGCTGGAACTGGTATTGCAGAGCTTATAGCCCTTGAAGTATCAAAACAGACAAATGCTCCAGTTGAGGAGACGCGCAA   1100
Consensus     gatcacac ttcttgttccttggtgctggggaagctggaactggtattgcagagcttatagcccttgaagtatcaaaacagacaaatgctccagttgaggagacgcgcaa

GX-19.txt     GAAGATTTGGCTTGTTGACTCAAAGGGATTAATTGTCCACTCGCGTAAGGATTCACTTCAACACTTCAAGACGCCATGGGCTCATGAGCACGAACCTGTCGGCGATTTAT   1210
SL-7.txt      GAAGATTTGGCTTGTTGACTCAAAGGGATTAATTGTCCACTCGCGTAAGGATTCACTTCAACACTTCAAGACGCCATGGGCTCATGAGCACGAACCTGTCGGCGATTTAT   1210
XDJQ-1.txt    GAAGATTTGGCTTGTTGACTCGAAGGGATTAATTGTCCACTCGCGTAAGGATTCCCTTCAACACTTCAAGAAGCCATGGGCTCATGAGCACGAACCTGTCGGCGATTTAT   1210
Consensus     gaagatttggcttgttgactc aagggattaattgtccactcgcgtaaggattc cttcaacacttcaaga gccatgggctcatgagcacgaacctgtcggcgatttat

GX-19.txt     TAAGTGCTGTCAAGGCAATTAAGCCAACAGTTTTGATTGGATCATCTGGAGTTGGTAGGACTTTTACAAAAGAAGTCATTGAGGCTGTGGCCTCCATCAATGAGAAACCT   1320
SL-7.txt      TAAGTGCTGTCAAGGCAATTAAGCCAACAGTTTTGATTGGATCATCTGGAGTTGGTAGGACTTTTACAAAAGAAGTCATTGAGGCTGTGGCCTCCATCAATGAGAAACCT   1320
XDJQ-1.txt    TAAGTGCTGTCAAGGCAATTAAGCCAACAGTTTTGATTGGATCATCTGGAGTTGGTAGGACTTTTACAAAAGAAGTCATTGAGGCTGTGGCCTCCATCAATGAGAAACCT   1320
Consensus     taagtgctgtcaaggcaattaagccaacagttttgattggatcatctggagttggtaggacttttacaaaagaagtcattgaggctgtggcctccatcaatgagaaacct

GX-19.txt     CTTATTATGGCTCTTTCCAACCCAACGTCACAATCTGAATGCACCGCTGAAGAGGCTTACACTTGGAGTGAGGGCCGTGCAATCTTTGCCAGTGGAAGTCCATTCGATCC   1430
SL-7.txt      CTTATTATGGCTCTTTCCAACCCAACGTCACAATCTGAATGCACCGCTGAAGAGGCTTACACTTGGAGTGAGGGCCGTGCAATCTTTGCCAGTGGAAGTCCATTCGATCC   1430
XDJQ-1.txt    CTTATTATGGCTCTTTCCAACCCAACGTCACAATCTGAATGCACCGCTGAAGAGGCTTACACTTGGAGTGAGGGCCGTGCAATCTTTGCCAGTGGAAGTCCATTCGATCC   1430
Consensus     cttattatggctctttccaacccaacgtcacaatctgaatgcaccgctgaagaggcttacacttggagtgagggccgtgcaatctttgccagtggaagtccattcgatcc

GX-19.txt     ATTTGAATACAACGGAAAGACCTTTGTCCCTGGCCAGTCCAACAATGCCTACATCTTCCCTGGATTCGGTTTGGGTGTCGTAATTTCTGGAGCAATTCGTGTGCACGACG   1540
SL-7.txt      ATTTGAATACAACGGAAAGACCTTTGTCCCTGGCCAGTCCAACAATGCCTACATCTTCCCTGGATTCGGTTTGGGTGTCGTAATTTCTGGAGCAATTCGTGTGCACGACG   1540
XDJQ-1.txt    ATTTGAATACAACGGAAAGACCTTTGTCCCTGGCCAGTCCAACAATGCCTACATCTTCCCTGGATTCGGTTTGGGTGTCGTAATTTCTGGAGCAATTCGTGTGCACGACG   1540
Consensus     atttgaatacaacggaaagacctttgtccctggccagtccaacaatgcctacatcttccctggattcggtttgggtgtcgtaatttctggagcaattcgtgtgcacgacg

GX-19.txt     ACATGCTTCTGGCTGCCTCTGAAGCATTGGCTGCCCAAGTCTCTGACGAGAACTACGACAAGGGATTGATCTACCCACCCTTTACTAATATCCGAAAGATCTCGGCAAAT   1650
SL-7.txt      ACATGCTTCTGGCTGCCTCTGAAGCATTGGCTGCCCAAGTCTCTGACGAGAACTACGACAAGGGATTGATCTACCCACCCTTTACTAATATCCGAAAGATCTCGGCAAAT   1650
XDJQ-1.txt    ACATGCTTCTGGCTGCCTCTGAAGCATTGGCTGCCCAAGTCTCTGACGAGAACTACGACAAGGGATTGATCTACCCACCCTTTACTAATATCCGAAAGATCTCGGCAAAT   1650
Consensus     acatgcttctggctgcctctgaagcattggctgcccaagtctctgacgagaactacgacaagggattgatctacccaccctttactaatatccgaaagatctcggcaaat

GX-19.txt     ATTGCAGCCAATGTTGCTGCCAAAGCATATGAACTTGGCTTGGCAACCCGTCTCCCGCGCCCTGCAGATCTTGTCAAGTATGCTGAGAGCTGCATGTACAGCCCCGTCTA   1760
SL-7.txt      ATTGCAGCCAATGTTGCTGCCAAAGCATATGAACTTGGCTTGGCAACCCGTCTCCCGCGCCCTGCAGATCTTGTCAAGTATGCTGAGAGCTGCATGTACAGCCCCGTCTA   1760
XDJQ-1.txt    ATTGCAGCCAATGTTGCTGCCAAAGCATATGAACTTGGCTTGGCAACCCGTCTCCCGCGCCCTGCAGATCTTGTCAAGTATGCTGAGAGCTGCATGTACAGCCCCGTCTA   1760
Consensus     attgcagccaatgttgctgccaaagcatatgaacttggcttggcaacccgtctcccgcgccctgcagatcttgtcaagtatgctgagagctgcatgtacagccccgtcta

GX-19.txt     CCGAACCTACCGATA   1775
SL-7.txt      CCGAACCTACCGATA   1775
XDJQ-1.txt    CCGAACCTACCGATA   1775
Consensus     ccgaacctaccgata
```

**Supplementary Figure S1.** Comparison of CDS sequences of Bch02G016960 of GX-19, SL-7 and XDJQ-1.

```
GX-19.txt   MESTLKEIGDGGSVLDLDPKATVGGGVEDIYGEDCATEEQLVTPWTFSVASGYSLLRDPHHNKGLAFTEKERDAHYLRGLLPPAIVTQQLQEKKLMQNIR    100
GX-71.txt   MESTLKEIGDGGSVLDLDPKATVGGGVEDIYGEDCATEEQLVTPWTFSVASGYSLLRDPHHNKGLAFTEKERDAHYLRGLLPPAIVTQQLQEKKLMQNIR    100
MY-1.txt    MESTLKEIGDGGSVLDLDPKATVGGGVEDIYGEDCATEEQLVTPWTFSVASGYSLLRDPHHNKGLAFTEKERDAHYLRGLLPPAIVTQQLQEKKLMQNIR    100
Consensus   mestlkeigdggsvldldpkatvgggvediygedcateeqlvtpwtfsvasgysllrdphhnkglaftekerdahylrgllppaivtqqlqekklmqnir

GX-19.txt   QYQLPLQKFIAMMELQERNERLFYKLLIDNVEELLPVVYTPTVGEACQKYGSIFRRPQGLYISLKEKGKILEVLKNWPQRSIQVIVVTDGERILGLGDLG    200
GX-71.txt   QYQLPLQKFIAMMELQERNERLFYKLLIDNVEELLPVVYTPTVGEACQKYGSIFRRPQGLYISLKEKGKILEVLKNWPQRSIQVIVVTDGERILGLGDLG    200
MY-1.txt    QYQLPLQKFIAMMELQERNERLFYKLLIDNVEELLPVVYTPTVGEACQKYGSIFRRPQGLYISLKEKGKILEVLKNWPQRSIQVIVVTDGERILGLGDLG    200
Consensus   qyqlplqkfiammelqernerlfykllidnveellpvvytptvgeacqkygsifrrpqglyislkekgkilevlknwpqrsiqvivvtdgerilglgdlg

GX-19.txt   CQGMGIPVGKLSLYTALGGVRPSACLPITIDVGTNNEKLLNDEFYIGLKQRRATGEEYYELLDEFMTAVKQNYGEKVLVQFEDFANHNAFELLAKYRTTH    300
GX-71.txt   CQGMGIPVGKLSLYTALGGVRPSACLPITIDVGTNNEKLLNDEFYIGLKQRRATGEEYYELLDEFMTAVKQNYGEKVLVQFEDFANHNAFELLAKYRTTH    300
MY-1.txt    CQGMGIPVGKLSLYTALGGVRPSACLPITIDVGTNNEKLLNDEFYIGLKQRRATGEEYYELLDEFMTAVKQNYGEKVLVQFEDFANHNAFELLAKYRTTH    300
Consensus   cqgmgipvgklslytalggvrpsaclpitidvgtnneklndefyiglkqrratgeeyyelldefmtavkqnygekvlvqfedfanhnafellakyrtth

GX-19.txt   LVFNDDIQGTAAVVLAGAVSALKLIGGTLADHTFLFLGAGEAGTGIAELIALEVSKQTNAPVEETRKKIWLVDSKGLIVHSRKDSLQHFKTPWAHEHEPV    400
GX-71.txt   LVFNDDIQGTAAVVLAGAVSALKLIGGTLADHTFLFLGAGEAGTGIAELIALEVSKQTNAPVEETRKKIWLVDSKGLIVHSRKDSLQHFKTPWAHEHEPV    400
MY-1.txt    LVFNDDIQGTAAVVLAGAVSALKLIGGTLADHTFLFLGAGEAGTGIAELIALEVSKQTNAPVEETRKKIWLVDSKGLIVHSRKDSLQHFKKPWAHEHEPV    400
Consensus   lvfnddiqgtaavvlagavsalkliggtladhtflflgageagtgiaelialevskqtnapveetrkkiwlvdskglivhsrkdslqhfk pwahehepv

GX-19.txt   GDLLSAVKAIKPTVLIGSSGVGRTFTKEVIEAVASINEKPLIMALSNPTSQSECTAEEAYTWSEGRAIFASGSPFDPFEYNGKTFVPGQSNNAYIFPGFG    500
GX-71.txt   GDLLSAVKAIKPTVLIGSSGVGRTFTKEVIEAVASINEKPLIMALSNPTSQSECTAEEAYTWSEGRAIFASGSPFDPFEYNGKTFVPGQSNNAYIFPGFG    500
MY-1.txt    GDLLSAVKAIKPTVLIGSSGVGRTFTKEVIEAVASINEKPLIMALSNPTSQSECTAEEAYTWSEGRAIFASGSPFDPFEYNGKTFVPGQSNNAYIFPGFG    500
Consensus   gdllsavkaikptvligssgvgrtftkevieavasinekplimalsnptsqsectaeeaytwsegraifasgspfdpfeyngktfvpgqsnnayifpgfg

GX-19.txt   LGVVISGAIRVHDDMLLAASEALAAQVSDENYDKGLIYPPFTNIRKISANIAANVAAKAYELGLATRLPRPADLVKYAESCMYSPVYRTY              590
GX-71.txt   LGVVISGAIRVHDDMLLAASEALAAQVSDENYDKGLIYPPFTNIRKISANIAANVAAKAYELGLATRLPRPADLVKYAESCMYSPVYRTY              590
MY-1.txt    LGVVISGAIRVHDDMLLAASEALAAQVSDENYDKGLIYPPFTNIRKISANIAANVAAKAYELGLATRLPRPADLVKYAESCMYSPVYRTY              590
Consensus   lgvvisgairvhddmllaasealaaqvsdenydkgliyppftnirkisaniaanvaakayelglatrlprpadlvkyaescmyspvyrty
```

**Supplementary Figure S2.** Comparison of protein sequences of Bch02G016960 of GX-19, SL-7 and XDJQ-1.

**Supplementary Table S2.** Fifty wax gourd materials were used to verify InDel marker.

| Number | Inbred line | Phenotype | Genotype | Number | Inbred line | Phenotype | Genotype |
|---|---|---|---|---|---|---|---|
| P1 | SL-7 | High SSC | High SSC | 25 | YS-13 | Low SSC | Low SSC |
| P2 | XDJQ-1 | Low SSC | Low SSC | 26 | YS-41 | Low SSC | Low SSC |
| F1 | F1 of SL-7×XDJQ-1 | The middle type | Heterozygote | 27 | YO-24 | Low SSC | Low SSC |
| 1 | GF-71 | High SSC | High SSC | 28 | YO-61 | Low SSC | Low SSC |
| 2 | 7-2-1-2-1 | High SSC | High SSC | 29 | F-23 | Low SSC | Low SSC |
| 3 | LV-46 | High SSC | High SSC | 30 | YO-22 | Low SSC | Low SSC |
| 4 | 7-2-1-2-5-1 | High SSC | High SSC | 31 | TK-12 | Low SSC | Low SSC |
| 5 | GX-71 | High SSC | High SSC | 32 | YM-33 | Low SSC | Low SSC |
| 6 | 7-2-1-3 | High SSC | High SSC | 33 | YO-16 | Low SSC | Low SSC |
| 7 | 7-2-1-5 | High SSC | High SSC | 34 | YS-11 | Low SSC | Low SSC |
| 8 | GK-33 | High SSC | High SSC | 35 | YMY-31 | Low SSC | Low SSC |
| 9 | 7-2-1-4-1 | High SSC | High SSC | 36 | YO-82 | Low SSC | Low SSC |
| 10 | 7-2-2-1-3 | High SSC | High SSC | 37 | YO-14 | Low SSC | Low SSC |
| 11 | B-1-1 | High SSC | High SSC | 38 | YS-45 | Low SSC | Low SSC |
| 12 | 7-2-1-4-5 | High SSC | High SSC | 39 | KF-23 | Low SSC | Low SSC |
| 13 | YM-33 | High SSC | High SSC | 40 | YS-11 | Low SSC | Low SSC |
| 14 | LV-6-8 | High SSC | High SSC | 41 | YO-23 | Low SSC | Low SSC |
| 15 | LV-21 | High SSC | High SSC | 42 | GD-1 | Low SSC | High SSC |
| 16 | YM-34 | High SSC | High SSC | 43 | GM-72 | Low SSC | High SSC |
| 17 | YM-13 | High SSC | High SSC | 44 | Temao11 | Low SSC | High SSC |
| 18 | GK-23 | High SSC | High SSC | 45 | HT-21 | Low SSC | High SSC |
| 19 | 7-2-1-5 | High SSC | High SSC | 46 | HK-43 | Low SSC | High SSC |
| 20 | GK-31 | High SSC | High SSC | 47 | F-23 | Low SSC | High SSC |
| 21 | 2hao | Low SSC | Low SSC | 48 | 3hao | Low SSC | High SSC |
| 22 | YM-24-1 | Low SSC | Low SSC | 49 | GD-23 | Low SSC | High SSC |
| 23 | YMY-16 | Low SSC | Low SSC | 50 | baidonggua | Low SSC | High SSC |
| 24 | YO-63 | Low SSC | Low SSC | | | | |
